# A young man with out-of-hospital cardiac arrest—it goes round and round

**DOI:** 10.1007/s12471-020-01480-4

**Published:** 2020-07-31

**Authors:** S. C. M. D. Panman, J. M. ter Maaten, Y. Blaauw

**Affiliations:** Department of Cardiology, University Medical Center Groningen, University of Groningen, Groningen, The Netherlands

A 24-year-old male patient presented at the emergency department after an out-of-hospital cardiac arrest. He had a witnessed arrest, after which basic life support was initiated by those present. An automated external defibrillator (AED), fetched by bystanders, delivered one shock, resulting in return of spontaneous circulation. His medical history involved palpitation complaints for which he visited the general practitioner’s practice. No definitive diagnosis had been made yet. His family history was clear of sudden cardiac death or cardiomyopathies. The electrocardiogram (ECG) at presentation at the emergency department is shown in Fig. 1. He was subsequently admitted to the cardiac care unit, where he experienced recurrent palpitations with stable haemodynamics. The ECG during palpitation complaints is shown in Fig. 2.

**Fig. 1:**
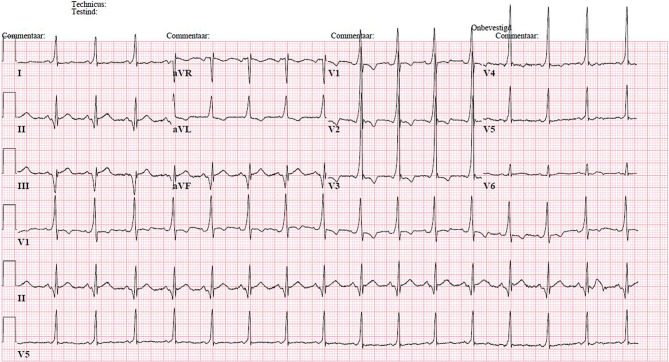
The ECG at presentation at the emergency department

**Fig. 2:**
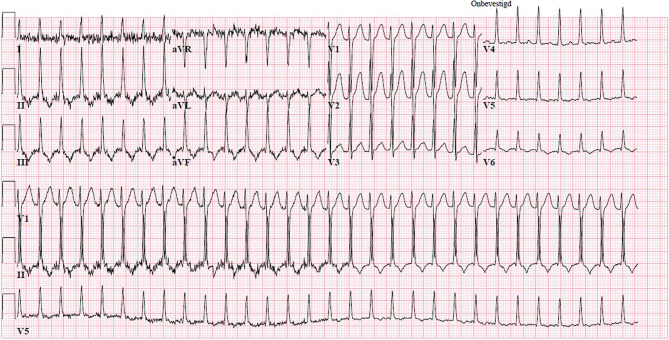
The ECG during palpitations complaints at the cardiac care unit

1. What is your diagnosis at this point?
2. What is the suggested therapy?

## Answer

You will find the answer elsewhere in this issue.

